# Supplementary figures and images for: Characteristics of Metabolites in the Development of Atherosclerosis in Tibetan Minipigs Determined Using Untargeted Metabolomics
Source: Nutrients. 2023 Oct 18;15(20):4425. doi: 10.3390/nu15204425 (PMC10609677; doi:10.3390/nu15204425)

Fig. S1

A

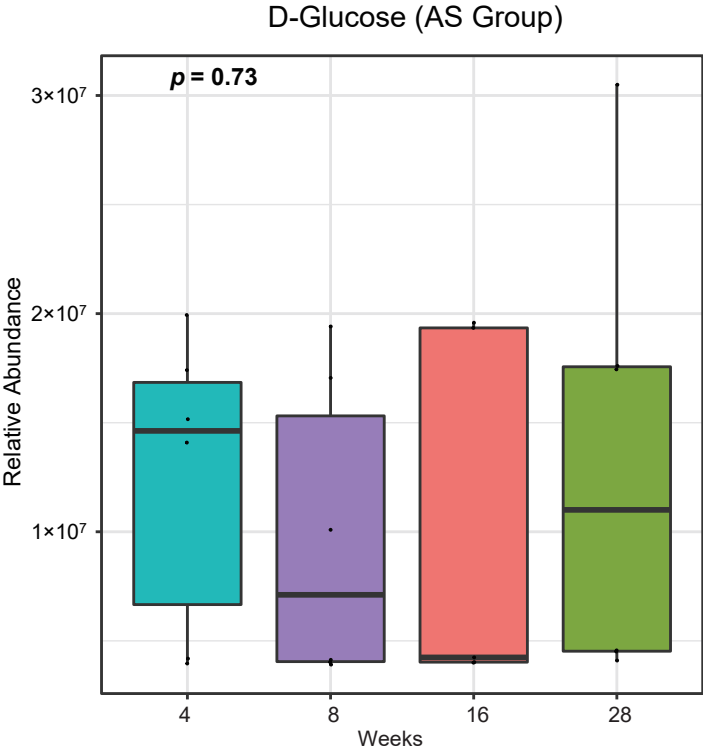

B

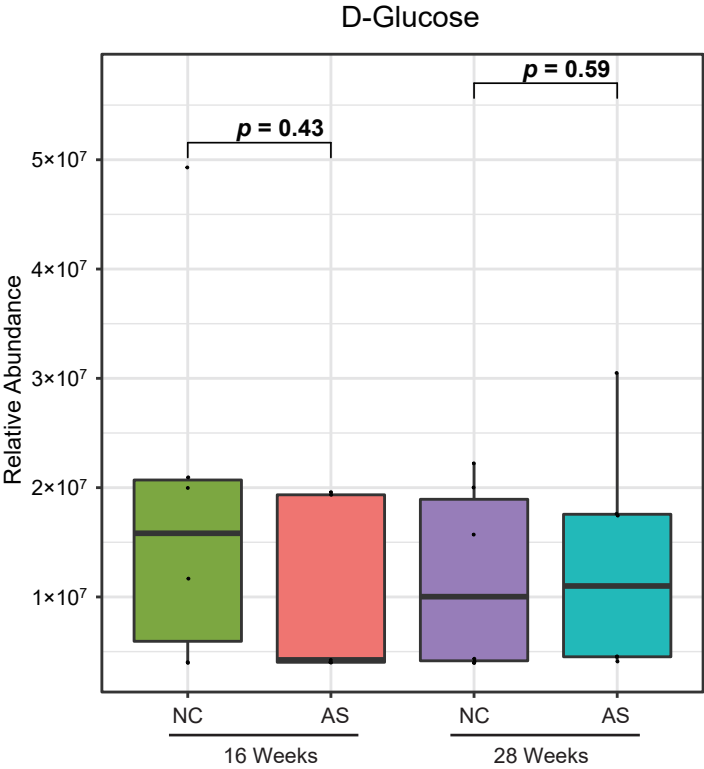

Fig. S2

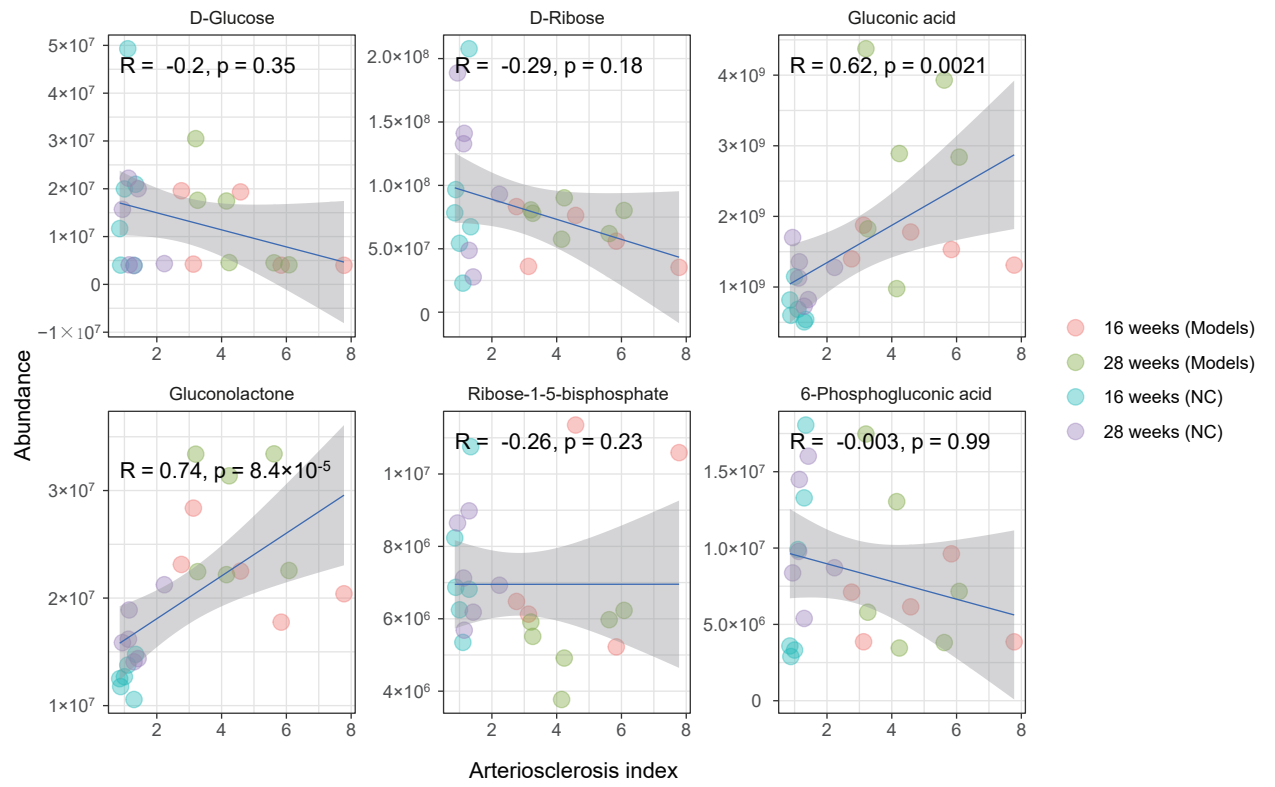

Fig. S3

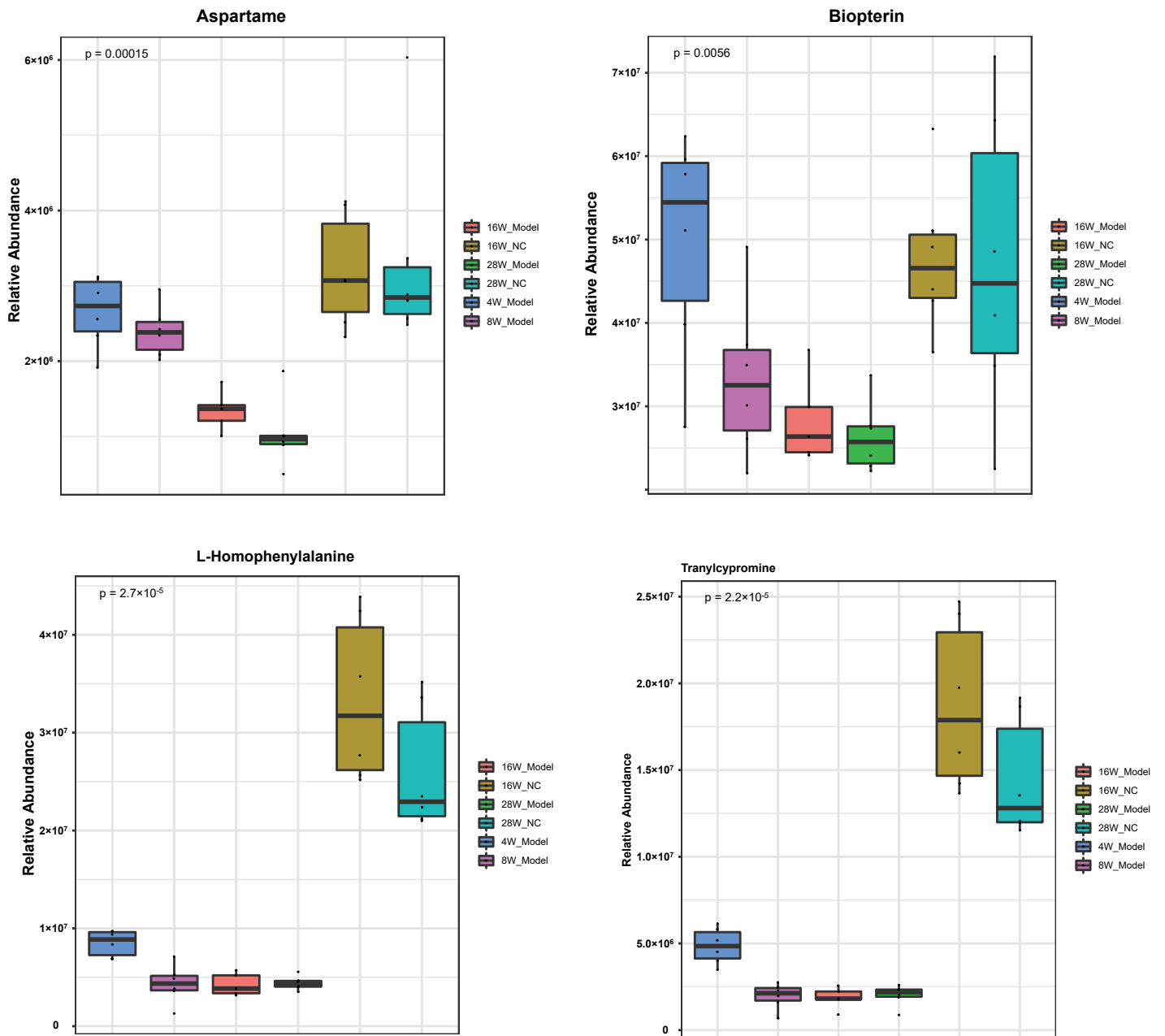

Fig. S4

A

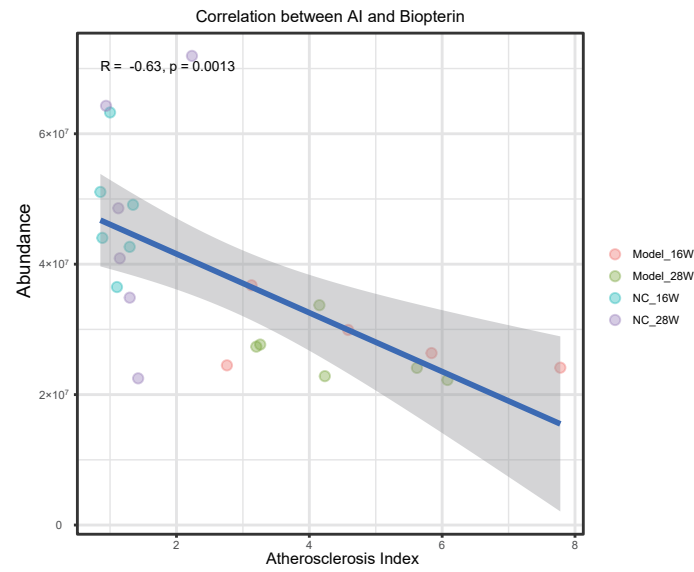

B

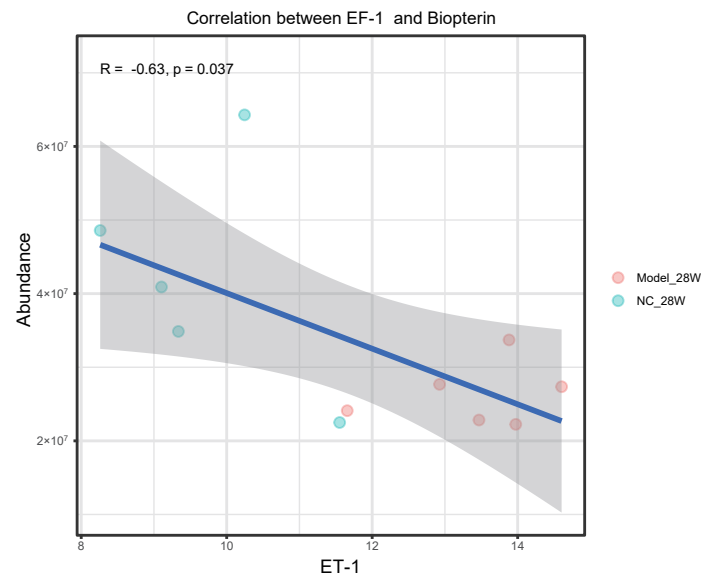

Supplement: Supplementary file 1 [file nutrients-15-04425-s001.zip › nutrients-2584272-supplementary.pdf]
